# Effects of Pandemic Response Measures on Crime Counts in English and Welsh Local Authorities

**DOI:** 10.1007/s12061-024-09614-6

**Published:** 2024-11-19

**Authors:** Niloufar Pourshir Sefidi, Amin Shoari Nejad, Peter Mooney

**Affiliations:** 1 Hamilton Institute, Maynooth University, Maynooth, Kildare Ireland; 2 Department of Computer Science, Maynooth University, Maynooth, Kildare Ireland

**Keywords:** Spatial statistics, Crime analysis, COVID-19 pandemic, England, Wales, Local authorities

## Abstract

The global response to the COVID-19 pandemic between January 2020 and late 2021 saw extraordinary measures such as lockdowns and other restrictions being placed on citizens’ movements in many of the world’s major cities. In many of these cities, lockdowns required citizens to stay at home; non-essential business premises were closed, and movement was severely restricted. In this paper, we investigate the effect of these lockdowns and other pandemic response measures on crime counts within the local authorities of England and Wales. Using openly accessible crime records from major police forces in the UK from 2015 to 2023, we discuss the impacts of lockdowns on the incidences of crime. We show that as time passed and citizens’ response to the imposed measures eased, most types of crime gradually returned to pre-pandemic norms whilst others remained below their pre-pandemic levels. Furthermore, our work shows that the effects of pandemic response measures were not uniform across local authorities. We also discuss how the findings of this study contribute to law enforcement initiatives.

## Introduction

During the COVID-19 pandemic, restrictions on the movement of citizens were enacted in almost every country worldwide to control the spread of the virus. The UK Government was no exception, implementing a series of extended measures in England and Wales beginning in March 2020. These lockdown-style policies have profoundly affected various sectors, including physical and mental health (Prati & Mancini, 2021; Mansfield et al., 2021; WHO, 2022), economic performance (Keogh-Brown et al., 2020), social interactions (Hussain et al., 2020; Calbi et al., 2021), mobility (Drake et al., 2020), and more. Recent studies underscore the significant impact of the pandemic and associated lockdowns on different types of crime globally (Halford et al., 2020; Langton et al., 2021a; Chen et al., 2021; Ceccato et al., 2022; Díaz-Faes et al., 2023). Investigating crime is a complex and multi-dimensional problem. Examining crime in relation to its spatial and temporal attributes helps researchers identify patterns, potentially illuminating the causes or influences behind crime in specific areas. This information is invaluable for shaping crime management policies within distinct regions and timeframes (Clancy et al., 2022). As we approach the three-year mark since the beginning of the COVID-19 pandemic, it is clear that lockdown-style policies have significantly altered people’s daily routines, leading to discernible shifts in crime patterns. While there is abundant research on the effects of government policies during the COVID-19 pandemic on crime (Nivette et al., 2021; Gerell et al., 2022; Hill et al., 2022), studies exploring the enduring post-pandemic effects and varied regional responses within England and Wales are scarce. This research aims to address this gap by analyzing the correlation between pandemic measures and crime counts in the local authorities of both England and Wales. Prior studies (Langton et al., 2021a, b; Neanidis & Rana, 2021; Farrell & Dixon, 2021; Dixon et al., 2021; Sun et al., 2021; Li et al., 2021; Frith et al., 2022; Trasberg & Cheshire, 2023) have explored UK crime patterns, these were mostly confined to the national level or focused solely on selected cities. Our study broadens this scope to encompass all local authorities. This expansion is important since trends at the national level could be predominantly driven by data from a few outlier regions. Additionally, studying crime at the local authority level offers a well-balanced and strategic perspective that bridges the gap between broader national analysis and highly specific street-level or neighbourhood data. At the local authority scale, it becomes possible to identify patterns and trends that are representative of larger and more diverse communities and encompass various environments and demographics. This level of analysis helps us avoid the potential interference and fluctuations that can obscure meaningful trends in extremely detailed analyses where day-to-day variations might overshadow significant patterns. Furthermore, local authorities typically bear the responsibility of resource allocation and policy agenda-setting for their entire jurisdiction. By examining crime at this level, decision-makers are in a better position to allocate resources, establish priorities, and develop strategies with a broad impact. In essence, while national level and more fine-grained analysis has great value, the local authority level provides an ideal framework for gaining a holistic understanding around implementing strategic measures in crime prevention and response. Consequently, this study focuses on three main research questions:

- **RQ1:** How have different crime types’ patterns evolved over time in the local authorities of England and Wales and have these patterns been influenced by the pandemic and its restrictions?
- **RQ2:** If there were changes in crime patterns during the pandemic, have these changes persisted post-pandemic?
- **RQ3:** What is the magnitude of the effect of restriction policies on the number of crimes within the local authorities for each crime type?

To address RQ1 we employ heatmaps to illustrate crime patterns over nine years, encompassing periods before, during, and after the pandemic. These heatmaps will assist in determining whether these trends are also evident in smaller, less populated regions. For RQ2, we reference the latest data from the UK police to assess whether different types of crime have returned to their pre-pandemic levels. Lastly, for RQ3, we construct a spatio-temporal regression model to estimate the effects of restriction intensity. We used the Stringency Index (Hale et al., 2021) as a proxy measure for restriction intensity in different local authorities.

The structure of the remainder of our paper is outlined as follows: in Section 2, we look at some of the most relevant literature pertaining to this domain. Section 3 outlines upon our data management approach, highlighting its limitations and the methodology adopted for this study. Our findings from the experimental analysis are summarised and discussed in Section 4. Lastly, the paper closes with Section 5 where the main contributions of our paper are outlined and some potential avenues for future research are described.

## Background and Related Work

In the first six months of the COVID-19 pandemic, Langton et al. (2021a) analyzed the alterations in different crime rates in England and Wales at an aggregate level of England and Wales as one. Their findings indicated that, with the exception of anti-social behaviour and drug-related crimes, there was a significant drop in all crime categories following the implementation of lockdown measures. However, as these restrictions were eased, many crimes saw an increase and it appears that residential burglaries might not resume their usual rates anytime in the near future. On the other hand Langton et al. reported that offences like robbery, violence and sexual offences did witness a swift return to their pre-pandemic levels. Similarly, Langton et al. (2021b) examined crime changes in England and Wales for the first seven months of the pandemic at the small-scale level. Their findings revealed that the drop in crime during the lockdown predominantly stemmed from a few specific areas, notably city centres with historically high crime rates. These trends can be attributed to the opportunity structure in those areas. However, even with the easing of lockdown restrictions, crime rates in these high-crime areas did not spike significantly and remained below the usual rates, even when areas with lower and medium crime rates reverted to their normal levels. In the study by Frith et al. (2022) the authors focused on crime data spanning from January 2019 to September 2020 to assess the pandemic’s influence on burglary crime within the London Metropolitan area at the borough level and hourly basis. The study uncovered a decrease in burglary incidents throughout the pandemic, particularly during the daytime. Halford et al. (2020) analyzed the different crime types (including violence and sexual offences, assault, public order, criminal damage and arson, vehicle theft and burglary) in one UK police force region, contrasting them with 5-year average rates. Their findings highlighted diverse timings for shifts in various crime categories; all except vehicle crimes began to decrease after the World Health Organization’s declaration of a global pandemic on March $11^{th}$ of 2020. By one week after the March $23^{rd}$ lockdown, there was a significant reduction in all crime categories, albeit with discrepancies. Halford et al. ; Al-Sabbagh et al. suggest that these changes in crime rates were predominantly influenced by changes in people’s movement patterns and locations respectively. In a study conducted by Neanidis and Rana (2021), the researchers explored how lockdown measures affected ten different categories of crimes, including burglary, criminal damage and arson, drugs, vehicle crimes, other thefts, possession of weapons, robbery, shoplifting, theft from the person, and violence and sexual offences, at the local authority level in England spanning from May 2013 to May 2021. Their research showed that, unlike localized lockdowns, nationwide lockdowns had a substantial impact on the trends in reported criminal activities, with the initial nationwide lockdown having the most noticeable effect. Furthermore, their investigation revealed that national lockdowns resulted in a significant decline in all the examined categories of crimes, except for anti-social behaviour and drug-related offences, which saw an increase. While Neanidis and Rana assessed the impact of lockdowns on various crimes across different local authorities, their work assumed a uniform effect for each lockdown across these regions. This approach has potential limitations such as overlooking spatial variations in response to the lockdowns.

Moreover, other studies have investigated the impact of COVID-19 on specific crimes through the lens of criminal theories, including Routine Activities Theory (RAT), General Strain Theory (GST) and Situational Action Theory (SAT). RAT (Cohen & Felson, 1979) suggests that crimes are not random occurrences but are instead either planned or opportunistic actions. Criminal events occur when three key elements converge in time and space: a motivated offender, a vulnerable target (such as a person or property seen as attractive to a potential offender), and a lack of guardianship (such as the limited presence of authority figures like police or school officials to deter such incidents). When all three elements are present simultaneously, the likelihood of a criminal event increases, whereas the absence of one factor reduces the opportunity for crime. The enforcement of COVID-19 containment policies and the practice of social distancing had repercussions on individuals’ activities, crime patterns, and emotional states. Research indicates that RAT is linked to a decline in certain crime types, such as thefts, robberies, homicides, and certain group-based offences (Boman & Gallupe, 2020; Campedelli et al., 2020; Mohler et al., 2020; Payne et al., 2020; Bullinger et al., 2021; Sun et al., 2021). General Strain Theory (GST) (Agnew, 1992) explains how encounters with stress can potentially result in acts of violence and criminal behaviour. According to GST, individuals may experience different types of stress, such as the failure to achieve valued goals, exposure to negative stimuli like abuse, or the removal of positive stimuli. These strains can trigger strong negative emotions and mental health issues. To cope with stress, individuals may employ various strategies, including emotional (e.g., substance abuse), cognitive (e.g., downplaying adversity), and behavioural (e.g., seeking social support or engaging in criminal behaviour) mechanisms. Under certain conditions, particularly when legitimate coping strategies are lacking, individuals are more likely to resort to violent and criminal behaviours. The COVID-19 pandemic has undoubtedly given rise to different strains and stresses. The implementation of lockdowns can be perceived as a negative influence. The loss of loved ones or financial difficulties also deprives individuals of positive stimuli. Recent research has delved into the impact of the pandemic’s stressful conditions, leading to an increase in mental health problems like depression and anxiety in both adolescents and adults, primarily due to heightened social isolation and stress (Magson et al., 2021; Coiro et al., 2021; McPherson et al., 2021; Zavlis et al., 2022; Cianfarani & Pampanini, 2023). Indeed, Zhang et al. (2020); Campedelli et al. (2021) have shown that the stress and negative emotions arising from the pandemic potentially contributed to an upsurge in violent crimes. Furthermore, it has been shown that there is a growth in intimate partner violence within many families due to the economic impact of the COVID-19 pandemic and the additional stressors introduced by social distancing measures (Zhang et al., 2020; Boman & Gallupe, 2020). Other studies have demonstrated that alcohol and drug usage has risen among both young individuals and adults as a strategy for dealing with the challenges posed by the pandemic (Dumas et al., 2020; Diaz-Martinez et al., 2021; Jacob et al., 2021; Villadsen et al., 2023). Situational Action Theory (SAT) (Wikström, 2014) explains the reasons behind criminal behaviour and, more broadly, why individuals adhere to or violate common rules of conduct. According to SAT, the causes of human actions are rooted in situational factors. People act the way they do based on their personal characteristics and the specific features of the environments they find themselves in. The theory also suggests that humans are fundamentally guided by rules and may commit a crime when they perceive it as an acceptable course of action given the situation, especially when no significant deterrents are present. Alternatively, individuals might engage in criminal behaviour when they fail to uphold their own moral standards (i.e., fail to exercise self-control) in situations where external pressures influence them to act against their own values (Wikström, 2017). Several studies have been conducted to examine the impact of COVID-19 pandemic policies and how these have altered situational environments, resulting in higher crime rates (Nivette et al., 2021; Campedelli et al., 2021; Ashby, 2020).

We believe that our research can provide additional support to the assertion that lockdowns in England and Wales significantly influenced the nature of criminal behaviour, as observed through the lenses of RAT, GST and SAT. Different local authorities possess varying demographics and socio-economic statuses, which can result in differing perceptions of injustice and levels of moral engagement. This leads to diverse levels of motivation for committing crimes due to factors like unemployment rates and the strictness of lockdowns. Moreover, there are varying levels of vulnerable targets and guardianship because of factors such as wealth. As discussed above, all of these factors could influence different crime patterns as predicted by RAT. Hence, we expect to see non-uniform impacts from interventions, such as lockdowns, on crime counts across the local authorities.

## Methodology and Approach

In this section, we provide an overview of the input datasets, including the crime dataset and the COVID-19 stringency index utilized in our study. We also elaborate on the methodology employed to assess the impact of lockdown strictness on crime.

### Study Area and Data

**Fig. 1:**
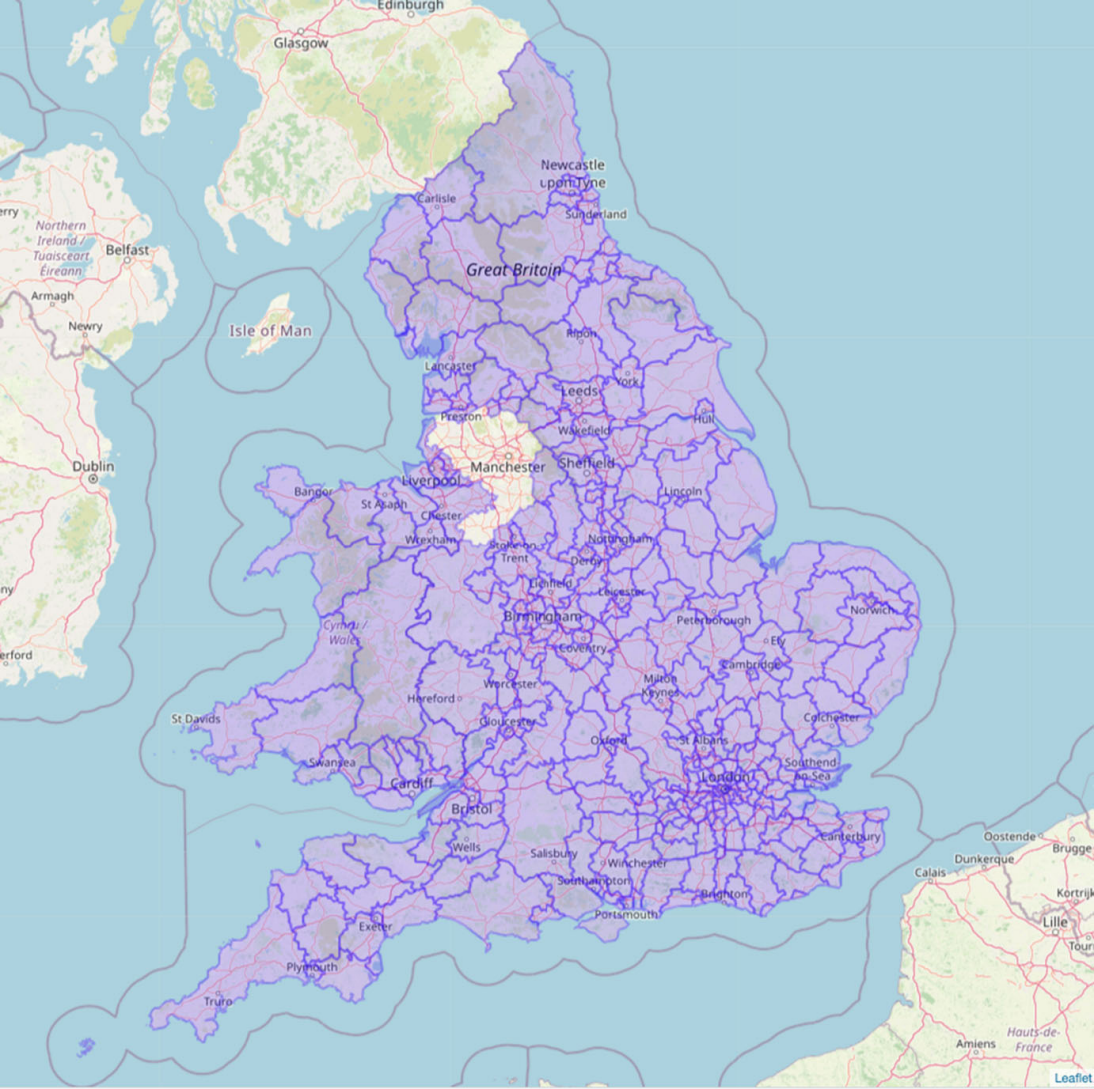
Map showing the study area of England and Wales, with local authorities outlined, excluding Greater Manchester due to data availability issues

**Fig. 2:**
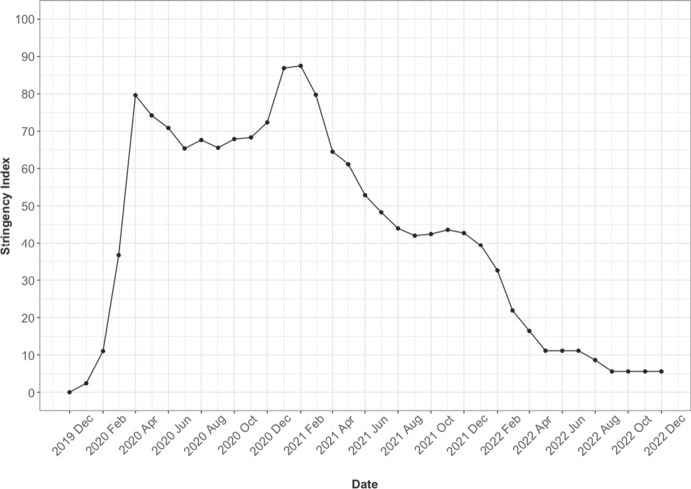
Variation in the stringency index (SI) in the UK between the years 2020 and 2022

England is geographically divided into 9 geographical regions with Wales being divided into 4 similar regions. These regions are further subdivided into a total of 339 lower-tier local authorities: 317 in England and 22 in Wales. These local authorities include London Boroughs, Unitary Authorities, and both Metropolitan and Non-Metropolitan Districts. In this study, we utilised two data sources as follows, both of which are applicable to England and Wales:

- **Crime data:** Each local authority in England and Wales falls under one of the 42 territorial police forces. These police forces record and report areal data on criminal activities on a monthly basis (Police UK, 2022). The spatial scope of the data is restricted to Lower Layer Super Output Areas (LSOA) which form a geographic hierarchy designed to enhance the reporting of small area statistics in England and Wales (National Health Service, 2022). For our study, we collected crime data^1^ spanning from January 2015 to May 2023. This period of time spans pre-pandemic, pandemic, and post-pandemic periods. We have aggregated the data to the local authority level. Table 1 provides a thorough summary of various crime categories, along with their respective explanations. Although the Police UK website https://data.police.uk/data/ offers the most detailed criminal reporting at a small-scale area throughout the UK, there are certain limitations within the period of our study. For instance, Greater Manchester Police has not submitted crime data to Police UK since July 2019 due to an IT system change. Additionally, crime data from Devon and Cornwall Police has not been made available since November 2022. There are more details on the Police UK website Police UK (2023). As a result, we have had to exclude local authorities in Greater Manchester entirely from our analysis. Additionally, our model employs crime data only up until November 2022, as all police forces, except for Greater Manchester, have at the time of writing finalized their reports up to that point (see Fig.  1).
- **Oxford Coronavirus Government Response:** The Oxford Stringency Index (SI) is a comprehensive measure designed to assess the strictness of government policies worldwide from 3rd January 2020 until 31st December 2022 (Hale et al., 2021). It encompasses nine response metrics, including school closures, workplace closures, cancellation of public events, restrictions on public gatherings, closures of public transport, stay-at-home requirements, public information campaigns, restrictions on internal movements, and international travel controls. Each day, the index is computed as the average score of these nine metrics for each country, with values ranging from 0 to 100. This provides a quantitative representation of the spectrum from the least to the most stringent restrictions. As noted by Cross et al. (2020), the SI fulfils the requirement for a standardized measure to compare the strictness of both national and international government responses across various days, and the index has gained widespread usage in assessing the impact of the COVID-19 pandemic and associated lockdowns on various problems, such as crime rates (Hill et al., 2022), stock market fluctuations (Yu & Xiao, 2023), COVID-19 mortality (Pourshir Sefidi et al., 2023), migration rates (Haslag & Weagley, 2022) and more. The methodology for calculating each metric is detailed in the work by Hale et al. (2021)^2^. We observed that the Oxford Coronavirus Government Response Tracker^3^ provided the daily SI for the entire UK, rather than reporting it separately for each country. Therefore, for the two countries under examination in this paper, we utilize the UK’s SI. To create monthly SI values for the UK, we computed the average index for each month, which aligned with the temporal resolution of our crime data. The changes in the SI, for the UK, from December 2019 to December 2022 are illustrated in Fig. 2.

**Table 1:** Crime categories from open police recorded crime data. See Police UK (2022) for more details

Crime category	Description
Anti-social behaviour	Personal, environmental and nuisance anti-social behaviour
Bicycle theft	Taking without consent or theft of a pedal cycle
Burglary	Person enters a house or other building with the intention of stealing
Criminal damage and arson	Damage to buildings and vehicles and deliberate damage by fire
Drugs	Offences related to possession, supply and production
Possession of weapons	Possession of a weapon, such as a firearm or a knife
Public order	Offences which cause fear, alarm or distress
Robbery	Offences where a person uses force or threat of force to steal
Shoplifting	Theft from shops or stalls
Theft from the person	Crimes that involve theft directly from the victim, without the use or threat of physical force (including handbag, wallet, cash, mobile phones)
Vehicle crime	Theft from or of a vehicle or interference with a vehicle
Violence and sexual offences	Offences against the person such as common assaults, grievous bodily harm and sexual offences

### Spatio-temporal Modelling

We utilize a Bayesian spatio-temporal model to evaluate different crime types over time and space within England’s major regions, as mentioned in Section 3.1. Our aim is to estimate the impact of restriction policies on the counts of various crime types within each local authority separately. The Bayesian modelling framework enables us to construct probabilistic models and appropriately quantify the uncertainty of estimated parameters using probability distributions. We adopted the model proposed by Bernardinelli et al. (1995), which has seen extensive use in various applications, notably in crime mapping (Luan et al., 2016). This model facilitates the modelling of area-specific trends, temporal trends, and the interactions between time and space and is fitted using the Integrated Nested Laplace Approximation (INLA) method as introduced by Rue et al. (2009). The R-INLA software (Lindgren & Rue, 2015) supports this model. To account for spatial and temporal dependencies, the Conditional Auto-regressive (CAR) model, as described by Besag (1974); Wall (2004), was employed. For our specific study, the model can be described as:

Let $Y_{i,j}$ represent the number of crimes at location $i = 1, \ldots , n$ and time $j = 1, \ldots , J$. The model can be expressed as:

1 $$\begin{aligned} Y_{i,j} \sim Poisson(\lambda _{i,j}) \end{aligned}$$

2 $$\begin{aligned} \log (\lambda _{i,j}) = \alpha + \eta _{q[j]} + (\beta + \delta _{i}) \times t_{j} + \gamma _{i} \times SI_{j} + u_{i} \end{aligned}$$

where:

- $\lambda _{i,j}$ is the time and area-specific expected value of crime.
- $\alpha$ is the intercept.
- $\eta _{q[j]}$ represents the effect of the quarter of the year at time $j$, capturing the seasonality effect.
- $\beta$ captures the overall temporal trend as the mean linear effect of time.
- $\delta _{i}$ denotes the space-time interaction effect.
- $\gamma _{i}$ represents the area-specific effect of the stringency index.
- $SI_{j}$ is the stringency index at time $j$.
- $u_{i}$ is the spatial random effect.

Bayesian models necessitate the definition of prior distributions for the model parameters. Upon fitting, these parameters are updated using the data, resulting in posterior distributions. In our model, we opted for the default prior distributions specified in INLA. Non-informative priors for the parameters $\alpha$ and $\beta$ are Gaussian with a mean of 0 and a variance of $10^{6}$. Hierarchical priors for $\delta _{i}$ and $\gamma _{i}$ follow $N(0, \sigma _{\delta }^{2})$ and $N(0, \sigma _{\gamma }^{2})$ independent and identically distributed (i.i.d) Gaussian distributions, respectively. For the spatial random effect $u_{i}$, we utilized the prior introduced by Besag et al. (1991), which is:

$$\begin{aligned} u_{i} \mid u_{z \ne i} \sim N\left( \frac{1}{n_{i}} \sum _{z \sim i} u_{z}, \frac{\sigma _{u}^{2}}{n_{i}}\right) \end{aligned}$$

where $z \sim i$ indicates all neighbors of region $i$, and $n_{i}$ is the total number of neighbors. $\sigma _{u}^{2}$ represents the variance of the spatial random effect. We used the default non-informative priors for the hyper-parameters defined in INLA as follows:

$$\begin{aligned} \log \frac{1}{\sigma _{\delta }^2}, \log \frac{1}{\sigma _{\gamma }^2}, \log \frac{1}{\sigma _{u}^2} \sim \log \gamma (1, 0.00005) \end{aligned}$$

## Result and Discussion

Since May 2022, the dedicated coronavirus team in the UK Government no longer exists (UK Government, 2022), due to changes in the pandemic situation reflecting shifts in the pandemic landscape such as rising population immunity through vaccination and prior infection alongside a decrease in the COVID-19 morbidity and mortality rate. This suggests a return of individuals in the UK to their usual routines and lifestyles. There is now a renewed focus on comprehending the spatio-temporal patterns of different crime types. As societies adjust to a post-pandemic world, understanding how crime patterns have evolved or remained constant during this period has become increasingly important.

**Fig. 3:**
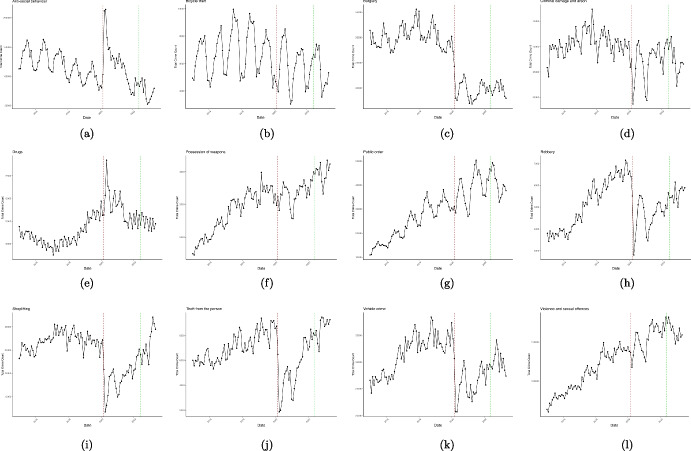
Temporal representation depicting diverse crime categories spanning the years 2015 to 2023 in England and Wales. The time series plots illustrate the total number of each crime type over the specified timeframe. The vertical red dashed lines denote the onset of the initial nationwide lockdown, while the green lines signify the conclusion of the pandemic

**Fig. 4:**
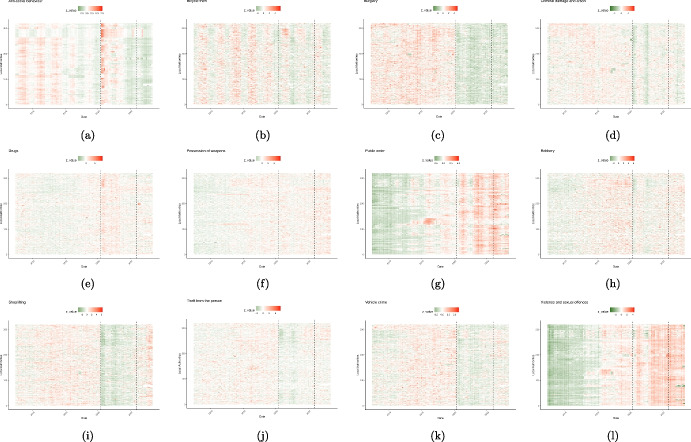
Spatio-temporal representation depicting diverse crime categories spanning the years 2015 to 2023 in England and Wales. The heatmaps depict the z-values associated with each crime type across varying local authorities and timeframes. Vertical dashed lines illustrate the commencement of the initial nationwide lockdown and the conclusion of the pandemic, respectively

**Fig. 5:**
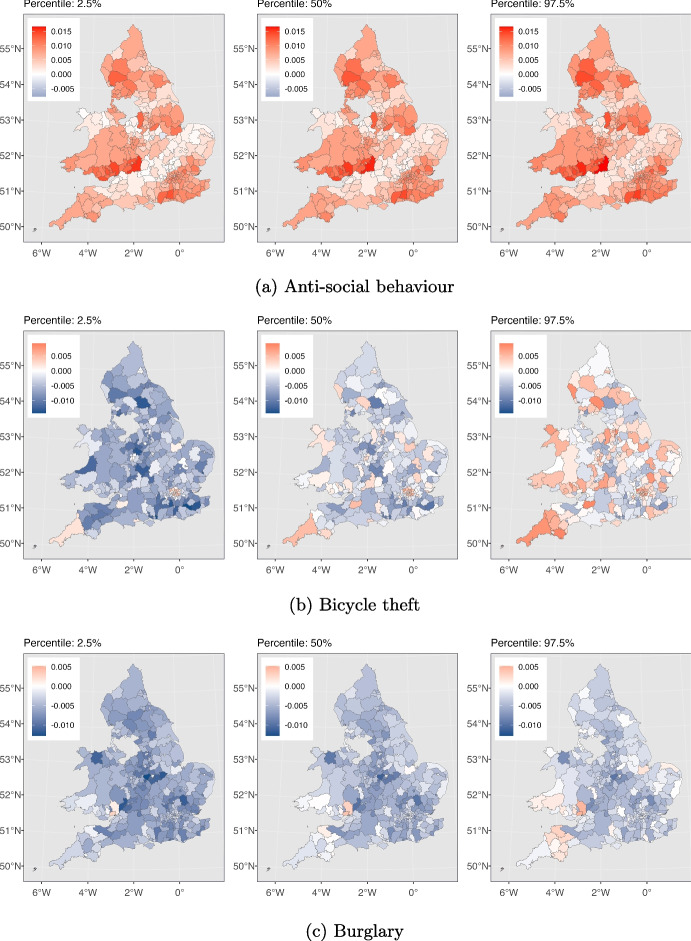
The effect of SI on different crime types in each local authority is depicted. For each crime type from left to right, the maps are associated with 2.5%, 50% and 97.5% cumulative probability for this effect, respectively. The numbers in the 2.5% and the 97.5% maps indicate the 95% credible interval for effects in each local authority


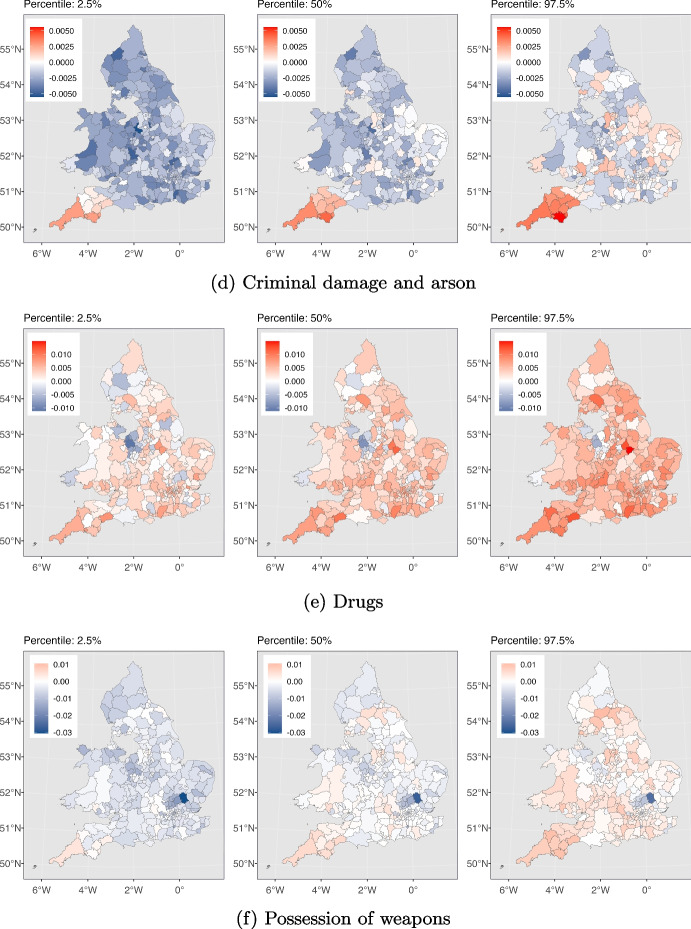



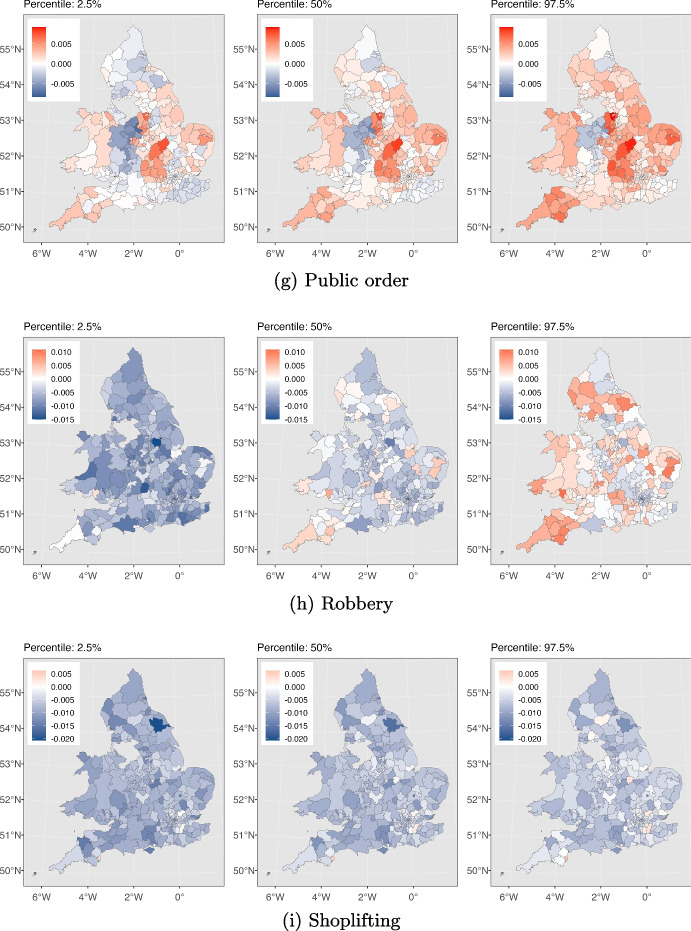



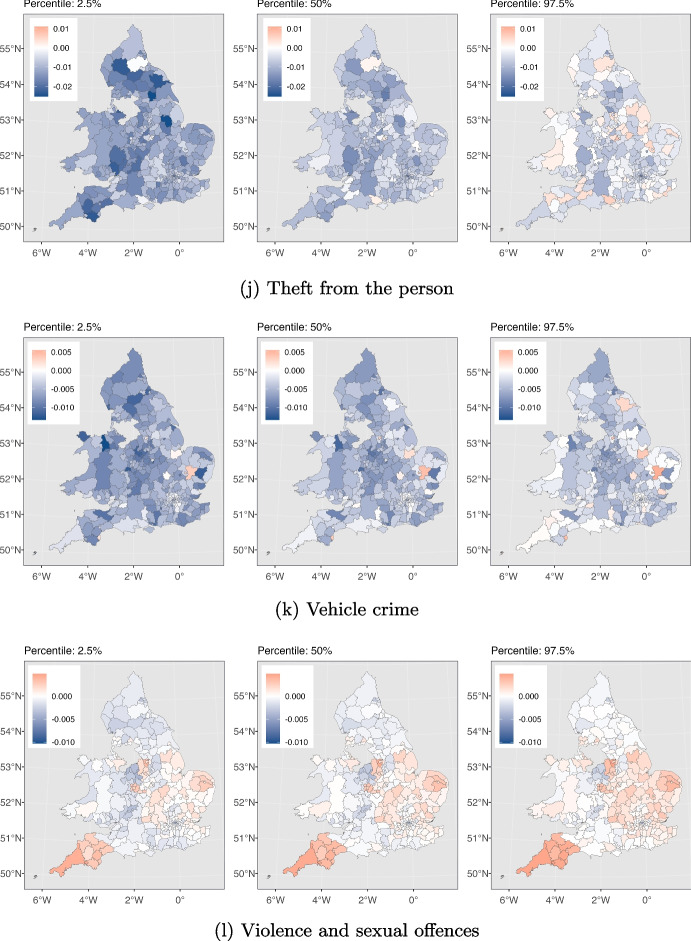


In this section, we first provide graphical representations of crime patterns in England and Wales for various crime types over a period of nine years (January 2015 to May 2023) at the local authority level in order to address RQ1 and RQ2. These visualizations show the crime data and illustrate yearly and seasonal fluctuations and geographical differences for each crime type. Our main goal is to explore crime trends before, during, and after the pandemic. To study these trends, we use two different visualizations: time series plots and heatmaps. Figure 3 displays time series plots that visualize the total count of specific crime types across England and Wales. For a deeper analysis of whether the crime trend is consistent among the majority of local authorities or is predominantly influenced by a few, we turn to heatmaps. Figure 4 displays the heatmaps, which offer a detailed representation of each crime across the 339 local authorities. The UK government issued a nine-character code, with one letter followed by eight numbers, to uniquely identify each local authority. Displaying all 339 local authorities with their codes on the heatmaps would be overwhelming and hard to read. To address this issue, we convert each local authority code into a unique number ranging from 1 to 339. This helps in simplifying the visualization. Moreover, local authorities that are neighbours and share borders have numbers that are close together. For instance, local authorities numbered 1 and 2 are neighbours. We scale the crime time series for each local authority to make them comparable for visualization purposes. As such, the heatmaps use z-values to present the crime data, providing insights into how crime counts differ from the average across regions and time. The colour intensity in each cell indicates the distance from the time series overall mean, with green showing negative z-values (below the average) and red showing positive z-values (above the average). Due to the absence of crime data from the Devon and Cornwall police force after November 2022, certain local authorities are represented in grey, indicating missing values, on the heatmaps for subsequent months. There are two distinct vertical dashed lines in each plot. These mark critical points in recent history: the start of the first national lockdown in March 2020 and the end of the pandemic in May 2022 in the UK which had notable impacts on crime trends.

We next delve deeper into understanding how different types of crimes were affected by the pandemic across various local authorities in England and Wales in order to address RQ3. We use maps to depict the influence of the stringency index on crime, as calculated by our model described in Section 3.2. Figure 5 displays these maps, highlighting the effects of stricter lockdown measures on each crime category throughout the study area. They also help us pinpoint local authorities that experienced a significant impact in the effects of the stringency index compared to others. As depicted in Fig. 5, for each crime category, we have presented three maps, from left to right, corresponding to the estimated stringency index effects at the 2.5%, 50%, and 97.5% cumulative probability levels. The values within the 2.5% and 97.5% maps represent the 95% credible interval for the stringency effect on that particular crime type within each local authority. Table 2 presents our model’s goodness of fit as measured by the $R^2$ and RMSE criteria, along with descriptive statistics for each type of crime. In the following sections, we discuss our findings for each crime type separately and explore potential drivers behind the observed patterns.

### Anti-social Behaviour Crime

Figure 3(a) shows the total instances of anti-social behaviour crimes spanning the period from January 2015 to May 2023. The plot exhibits seasonality, with consistent and predictable changes occurring every year. Notably, each year, the pattern exhibits an increase starting from March, peaking in July, and declining until December. We observe an overall downward trend with some fluctuations leading up to the period just before the pandemic. Then a conspicuous rise in the total count of anti-social behaviour crimes coincides with the onset of the COVID-19 pandemic (shown by the red dashed line, indicating March 2020). This suggests that the pandemic and the ensuing lockdown measures plausibly promoted anti-social behaviour offences, leading to a temporary increase. According to Fig. 4(a), the majority of local authorities have experienced similar patterns, demonstrating that our deductions are not solely based on a few regions. Furthermore, Fig. 5(a) illustrates the variation in the impact of the stringency index on anti-social behaviour crimes across different local authorities in England and Wales. This effect appears to be relatively consistent and positive, with some regions experiencing greater impacts.

**Table 2:** Offences categories and corresponding descriptive statistics, and the model’s goodness of fit for each category for January 2015 to May 2023

Crime category	Total count	Mean	Std.	$R^2$	RMSE
Anti-social behaviour	11461783	357.28	340.07	0.92	94.62
Bicycle theft	645018	20.96	29.40	0.87	10.37
Burglary	2634785	82.25	90.52	0.93	22.72
Criminal damage and arson	3972475	123.77	112.49	0.95	23.55
Drugs	1207719	37.73	46.79	0.90	14.22
Possession of weapons	307875	10.06	13.58	0.87	4.80
Public order	3015607	94.07	112.19	0.92	30.31
Robbery	514907	17.64	34.90	0.92	9.00
Shoplifting	2505011	78.24	77.66	0.93	19.96
Theft from the person	690501	23.13	79.19	0.92	20.57
Vehicle crime	3031008	94.57	115.54	0.94	27.35
Violence and sexual offences	12909980	401.61	412.53	0.96	78.63

For consistence, in column one, we use the same crime category names as used by Police UK

Subsequent to the initial surge (April and May 2020), a notable reduction in instances of anti-social behaviour crimes becomes apparent. The most pronounced reduction occurs between December 2020 and January 2021, coinciding with the period of the highest stringency index. Interestingly, despite the increasing impact of stricter lockdown measures during the initial increase, the higher stringency index in December and January does not lead to an increase in the overall count of anti-social behaviour crimes. This counteractive effect could potentially be attributed to the previously discussed seasonal trend, where the total count of this type of crime typically reaches its lowest point in December and January each year compared to other months. Subsequently, this declining trend persists beyond the conclusion of the pandemic (depicted by the green dashed line representing May 2022). Therefore, despite the initial surge in criminal activities brought about by the start of the pandemic, it is evident that this particular type of crime maintains its ongoing downward trajectory that has been established since 2015. The alteration in the trend of anti-social behaviour crimes during the onset of the pandemic can be attributed to certain individuals resisting the strict lockdown measures. Moreover, the pandemic placed considerable strains on people’s daily lives, including instances such as job losses, financial difficulties, the loss of loved ones, feelings of social isolation, concerns regarding vaccination, and a range of other pandemic-related anxieties (Magson et al., 2021; Coiro et al., 2021; McPherson et al., 2021; Zavlis et al., 2022; Cianfarani & Pampanini, 2023). These factors played a role with many individuals struggling with negative emotions. These emotions may have been intensified due to the lack of healthy coping mechanisms such as social interactions. This aligns with the core principles of the GST, as discussed in Section 2. Additionally, the heightened stress and anxiety due to financial and emotional difficulties led to moral disengagement, where some individuals justified anti-social behaviour as a reaction to their own perceived injustices or frustrations with pandemic restrictions. Shifts in social norms during this period may have further contributed to these behaviours, as some people perceived anti-social actions as more acceptable under extraordinary circumstances, which is consistent with the principles of SAT. After the restrictions were eased, the trend seems to be now reverting back to its pattern before the pandemic.

### Bicycle Theft

According to Fig. 3(b), a rising trend in bicycle theft is evident until 2018, followed by a subsequent decline. The presence of a noticeable seasonal pattern is also apparent, with certain months consistently showing higher rates of bicycle theft. This recurring pattern could be influenced by factors such as weather conditions (such as warmer months potentially leading to increased bicycle usage and subsequently more thefts), holidays, an influx of tourists, and other annual events. This aligns with RAT, suggesting that during periods of favourable conditions, the attractiveness of alternative transportation options like bikes and scooters increases, providing more opportunities for street criminals.

While temporary fluctuation can be observed - like a momentary reduction coinciding with the beginning of the pandemic (March 2020) unlike previous years these tend to balance out over the long run. When analyzing the longer period of nine years (2015-2023), the total count of bicycle thefts demonstrates a consistent decline post-2018. It appears that the pandemic did not alter the existing trend of bicycle theft observed prior to the pandemic. This suggests that, despite external influences and year-to-year variations, the broader pattern of bicycle thefts has remained relatively stable. This viewpoint contrasts with the conclusions drawn from some other studies (Langton et al., 2021a; Agrawal et al., 2022). While these studies have emphasized specific short-term increases in theft rates, our analysis offers a more holistic perspective by spanning the entire duration of the pandemic and its aftermath. Through this comprehensive examination, we have observed a persistent decrease in the overall trend since 2018. According to Fig. 4(b), this holds true for the majority of local authorities.

In Fig. 5(b), we depict the variation in the impact of the stringency index on bicycle theft crimes across local authorities in England and Wales. This effect shows notable disparities among the local authorities. Notably, certain areas, such as those within the City of London, exhibit significant positive values of this effect, while others exhibit significant negative values of the effect. This can be explained by the fact that there are more households in the city of London without access to a car or van compared to the rest of England and Wales (ONS, 2021). As a result, their primary transportation choices are limited to public transportation like buses and subways, taxis, or bicycles. During the lockdowns the government either partially suspended public transportation services or reduced their capacity (CRISIS24, 2020) and this encouraged more people to opt for bicycles. This shift in transportation preferences has altered the opportunity structure for bike thefts in central London.

### Burglary

Figure 3(c) shows no prominent trend from 2015 to 2019 in burglary incidences. However, in 2019, a declining trend begins to emerge, and this downward trajectory becomes more pronounced with the onset of the pandemic. Notably, there are two distinct declines since the start of the pandemic. The first is a substantial drop at the beginning of the pandemic followed by another drop in January/February 2021. These declines are aligned with periods when the stringency index reached its peak values. These drops can be attributed to the fact that more individuals were staying at home during these times likely resulting in fewer unattended targets for burglars. Figure 4(c) confirms that the pattern is fairly consistent across all local authorities.

However, burglary incidents have not reached pre-pandemic levels and remain significantly lower. Several factors could account for this new sustained trend: (1) increased residence occupancy: the adoption of remote work and extended periods spent at home due to the pandemic have reduced the opportunities for burglars which aligns with the concepts of RAT. (2) Elevated security measures: due to the effects of COVID-19, more people have been staying at home, prompting a heightened interest in fortifying their residences and purchasing security-related equipment they might have postponed previously. This surge in home security and CCTV system acquisitions is evident in the UK, where there is approximately one camera for every 13 individuals (Yong, 2018). Furthermore, this number is projected to escalate significantly from 2021 to 2025 by around 26% (White, 2021). Hence, homeowners, exhibiting increased security awareness during the pandemic, have embraced these upgraded security solutions. These measures continue to deter burglaries by decreasing the pool of susceptible targets, aligning with the principles of RAT, even in the post-pandemic phase.

The figures also unveil a seasonal pattern, with heightened burglary counts in specific months such as October and November, while counts are relatively lower from March to September. This could be attributed to various factors, such as increased shopping activities during October and November due to sales seasons like Black Friday, Christmas, and New Year, leading to more valuable items in homes. Additionally, the decrease in daylight hours during these months may offer cover for burglars who aim to avoid being seen. With respect to the effect of the stringency index, Fig. 5(c) displays the variation in the effect on burglary crimes in various local authorities in England and Wales. This effect seems to be uniform and negative across most local authorities. However, it is worth noting there are a few local authorities, namely Monmouthshire and Newport, that exhibit a positive effect. At the time of writing this paper, the reason for the positive effect in these regions is unclear to us and warrants further investigation, which we defer to future work.

### Criminal Damage and Arson

Figure 3(d) illustrates the total count of criminal damage and arson cases spanning from 2015 to 2023. During the pandemic period, there are two noticeable declines in the data, one occurring at the commencement of the pandemic (indicated by the red dashed line), and the other in February 2021. These drops coincide with the peaks of the stringency index and stricter lockdowns. Following the second drop we can see that the trend rebounds. Figure 4(d) illustrates that this behaviour occurred in the majority of the local authorities. It appears that the downward trajectory of criminal damage and arson crimes since late 2017 has persisted. The pandemic’s impact on this trajectory seems to be a temporary decrease rather than a lasting change.

Figure 5(d) demonstrates the variation in how the stringency index affects criminal damage and arson crimes in various local authorities across England and Wales. This effect seems to be negative and consistent across various local authorities. However, a minority of the local authorities changed colour from blue to red in the 2.5% and 97.5% panels respectively. This should caution us against drawing conclusions with high certainty about the impact of the stringency index in these areas.

### Drugs

As depicted in Fig. 3(e), the total count of drug crimes in England and Wales follows a fluctuating pattern, initially displaying a downward trend until the end of 2016. Subsequently, the trend reverses and begins to rise, gaining momentum by the onset of the pandemic, with a peak in May 2020. However, this surge subsides, and the trend begins to flatten, eventually aligning closely with its pre-pandemic levels from mid-2021 onward. This suggests that the pandemic and subsequent lockdown measures influenced the trajectory of drug-related offences, leading to an increase in such incidents. This increasing impact fades as pandemic measures are relaxed. Moreover, Fig. 4(e) reveals that drug crime exhibits either low seasonal fluctuations or none that are clearly discernible. This trend can be attributed to the majority of the local authorities.

Multiple factors might have contributed to the heightened levels of drug-related crimes during the initial year of the pandemic. These factors are outlined as follows: (1) Psychological well-being: the pandemic’s impact on mental health could has contributed to heightened drug consumption and this subsequent increase (Magson et al., 2021; Coiro et al., 2021; McPherson et al., 2021; Zavlis et al., 2022; Cianfarani & Pampanini, 2023) aligns with the principles of GST, RAT and SAT. (2) Shift in law enforcement focus: law enforcement agencies might have redirected their attention to street-level activities during the pandemic’s early stages, leading to heightened proactive efforts in drug-related operations. This adjustment could have been facilitated by the increased visibility of dealers and suppliers, who encountered greater difficulty moving around freely due to the lockdown measures (Casciani & Butcher, 2021; Halford et al., 2022). Given that these two factors have almost reduced post-pandemic, this could explain why the trend in drug crimes has returned to its pre-pandemic pattern. According to Fig. 5(e), it is evident that the impact of the stringency index on drugs crime is somewhat uniform and positive in the majority of local authorities. Nevertheless, it is noteworthy that several local authorities in the North West of England and Pembrokeshire exhibit significant negative values of this effect.

### Possession of Weapons

Figure 3(f) displays a significant upward trajectory preceding the pandemic, with the bulk of the increase occurring between March 2016 and March 2019 (ONS, 2020). As the pandemic began, the trend shifted direction, resulting in the lowest point since mid-2017 in February 2021, coinciding with the highest value of the stringency index. This decline may be linked to the implementation of stricter lockdown measures, which shifted law enforcement’s attention towards public spaces. Consequently, there was an elevated risk for criminals to be apprehended by authorities for carrying weapons. This heightened risk aligns with the concepts of the RAT, leading to a decrease in the likelihood of this crime occurring. Starting in mid-2021, the government initiated a relaxation of some pandemic measures (for more detail, see Fig. 2). Subsequently, the trend for weapon possession crimes began to rise once more. This suggests that the pandemic’s effect on this crime category was not persistent. However, it is worth noting that, according to Fig. 4(f), there are different clusters of local authorities. Some peaked between 2016 and 2018, then experienced a decline. Many peaked between 2018-2019 and declined with the onset of the pandemic, never returning to pre-pandemic levels. In contrast, a significant number reached their peaks post-pandemic.

Figure 5(f) illustrates the impact of the stringency index on weapon possession crimes in local authorities across England and Wales. As shown in the figure, numerous local authorities shifted from blue to red in the 2.5% and 97.5% plots, respectively. This includes a zero-effect interval, meaning we cannot definitively claim whether the effect has been positive or negative for these regions. Conversely, in certain regions like Uttlesford (England) the effect is notably negative, while in places like Neath Port Talbot (Wales), it is significantly positive.

### Public Order

Figure 3(g) presents the total count of public order crimes over a span of nine years and there is a discernible upward movement in the trend. This could potentially be attributed to economic difficulties and mental well-being (Cribb et al., 2021; MCrae et al., 2023; Slawson, 2021). Additionally, the plot shows a clear seasonal pattern with certain months consistently exhibiting elevated occurrences of public order crimes, such as June and July, while other months show a decrease, including the period from December to February. This seasonality could potentially be attributed to various factors, including notable public events like Black Lives Matter rallies and far-right counter protests (during June and July) (Grierson, 2020), as well as holiday seasons like Christmas and New Year when individuals often opt to stay indoors with their families, participating in seasonal celebrations. This shift towards indoor activities results in fewer outdoor engagements and gatherings and fewer opportunities for conflicts or disruptions that could potentially escalate into public order crimes.

The indicated upward trend experiences two declines. There is one minor decline at the beginning of the pandemic and another decline in January/February 2021. These drops coincide with the periods when the stringency index reached its peak, indicating that the implementation of lockdowns had an effect on the occurrence of public order crimes. However, as time progressed and certain individuals disregarded government mandates, the count rose once again. This pattern is consistent with the principles of the GST, where individuals when confronted with these strains, opt for resistance against the government and causing fear and distress. Also, heightened stress and anxiety due to the pandemic, along with perceptions of injustice and moral disengagement, may have led individuals to justify their criminal behaviour as a reaction to their perception of overreaction by authorities, which aligns with the principles of SAT. Additionally, according to Fig. 4(g), this pattern is evident for most local authorities, with only a small number of local authorities diverging from the mainstream trend. Overall, public order crimes have been persistently higher than pre-pandemic levels. This cannot necessarily be attributed solely to the lockdowns, as the rising trend predating the pandemic is also a factor. According to Fig. 5(g), the effect of the stringency index on public order crimes in the local authorities of England and Wales is heterogeneous. The effect has been positive in many areas such as East Midlands while being insignificant or negative in other places like Shropshire and Stafford.

### Robbery

Based on Fig. 3(h), the robbery trend demonstrates a rise until March 2019, followed by a decline leading up to the pandemic. Subsequently, there are two noticeable drops: one coinciding with the initiation of the pandemic (highlighted by the red dashed line), and the other in February 2021. These findings correspond with the peaks of the stringency index and indicate a noticeable impact of the pandemic and its associated restrictions on the trend of robbery. Thereafter, the trend shows a partial rebound, and it continues to move in an upward trajectory. However, the levels post-pandemic have not managed to reach the heights observed in previous years. Figure 4(h) shows that this pattern holds true for the majority of the local authorities, and it is not dominated by a few outliers.

Several factors could explain the persistent lower number of robbery crimes even after the pandemic: (1) Shift in opportunities: the prevalence of remote work may result in fewer opportunities for street-level robberies due to reduced foot traffic. (2) Heightened security measures: the increased installation of CCTV cameras, alarm systems, and improved lighting in public spaces and residential neighbourhoods (Yong, 2018; White, 2021; WLC, 2021), could serve as deterrents, dissuading potential robbers and thus contributing to the decline in robbery crimes. There are some linkages to the discussion around burglary in Section 4.3.

The impact of the stringency index on robbery crime throughout England and Wales is presented in Fig. 5(h). According to the figure, the effect is negative with at least a 50% probability for the vast majority of the local authorities. However, examining the panel from left to right, we notice that many regions have shifted from blue to red. This shift indicates a non-negligible probability that the effect could be positive, which cautions against drawing definitive conclusions about the magnitude of the effect in those regions. Nonetheless, there is only one region, which is Neath Port Talbot (Wales), that exhibits a significant positive effect.

### Shoplifting

Figure 3(i) reveals alternating phases of upward and downward trends in the shoplifting crime pattern prior to the pandemic, with the upward trend persisting until the end of 2017. Subsequently, the downward trend that commenced at the end of 2017 continues, marked by a significant drop at the onset of the pandemic. An additional decrease is noticeable in January/February 2021. These declines are likely attributable to the closure of numerous businesses and reduced in-person shopping. Consequently, it is reasonable to expect that opportunities for shoplifting diminished during this period and this observation aligns with the principles of RAT. After this decrease, the trend begins to rise, eventually surpassing the levels observed before the pandemic. Furthermore, Fig. 4(i) indicates that this trend is consistent across the majority of local authorities. Similarly, Fig. 5(i) illustrates consistent negative values in the impact of the stringency index on shoplifting crimes throughout England and Wales except for Sevenoaks and Gosport (both England) which experienced significantly positive impacts.

### Theft from the Person

Figure 3(j) illustrates a general upward trend in these incidents throughout the years until the advent of the pandemic. Two distinct drops in the pattern are apparent: one taking place at the beginning of the pandemic (marked by the red dashed line), and the other in January/February 2021, corresponding with the highest points of the stringency index. These drops can be attributed to the pandemic and the enforced lockdowns altering people’s routine activities. With many individuals confined to their homes, limitations on public gatherings, and the closure of non-essential businesses, the opportunities for thieves have significantly diminished on the streets and in public areas. Following the mentioned pronounced declines, incidents of theft from the person began to rise once more, eventually reaching levels similar to those observed before the pandemic. This suggests that the alteration in the pattern of theft from the person crimes during the pandemic was not sustained which is consistent with the RAT.

Looking at Fig. 4(j), we do not see evidence of a few regions dominating the overall trend. Instead, the majority of the local authorities exhibit similar patterns. Figure 5(j) displays the effect of the stringency index on theft from the person crimes throughout England and Wales. This effect seems to be negative and consistent across various local authorities. However, a minority of the local authorities changed colour from blue to red in the 2.5% and 97.5% panels respectively. This should caution us against drawing conclusions with high certainty about the impact of the stringency index in these areas.

### Vehicle Crime

As depicted in Fig. 3(k), there is an increasing trend observed until 2019, followed by a subsequent decline. Two distinct drops in the trend of vehicle crimes are evident - one at the beginning of the pandemic (marked by the red dashed line), and another in February 2021, coinciding with the highest points of the stringency index. This suggests that the pandemic and the associated lockdowns had a substantial impact on vehicle crimes. As time progresses, the trend begins to rise again. However, the crime count in the post-pandemic period has not reached the levels observed prior to the pandemic’s outbreak. According to Fig. 4(k), this pattern is not uniform across all local authorities. There is noticeable heterogeneity; some local authorities have recovered their pre-pandemic levels more than others. The ongoing decrease in vehicle crime after the pandemic in certain areas can be attributed to several factors such as:

(1) Diminished vehicle utilization: With more individuals continuing to work from home in these areas post-pandemic, there is a reduction in the number of vehicles on the road, leading to fewer opportunities for theft.

(2) Heightened security measures: The increased deployment of CCTV cameras, coupled with improved lighting in public areas and residential neighbourhoods, elevates the risk of criminals being apprehended.

Furthermore, Fig. 3(k) showcases a seasonal pattern. Specific months consistently exhibit heightened occurrences of vehicle crimes, while others show a decrease. This cyclic pattern could potentially be influenced by various factors such as weather. For instance, in colder months it is plausible that people might be more likely to use their vehicles for commuting due to unfavourable weather conditions. This increased vehicular activity could lead to higher instances of vehicle-related crimes. There are more cars on the road and parked in various locations. Holiday periods can see people travel and vehicles are left unattended, or other seasonal influences that are in accordance with the concepts outlined in RAT. In Fig. 5(k), we present the impact of the stringency index on vehicle-related crimes across England and Wales. This impact is predominantly negative and consistent across most local authorities. However, West Suffolk (England) is an exception where the effect has been positive.

### Violence and Sexual Offences

According to Fig. 3(l), the total count of violence and sexual offences in England and Wales shows an overall ascending trend from 2015 to 2023. The upward trend observed over the years could be explained by several factors. These include a greater willingness of victims to report such crimes, including those from the past, along with improvements in the recording procedures by law enforcement (ONS, 2021). Additionally, the surge in stress, anxiety, and challenges related to mental health (Campbell, 2020) might contribute to an increased propensity for violent behaviours (Mohler et al., 2020; Piquero et al., 2021). This alignment with the GST and SAT suggests that these external pressures might intensify the likelihood of engaging in aggressive actions.

Two noticeable declines in the trend are observable, one occurring at the onset of the pandemic (indicated by the red dashed line), and the second one in February 2021, aligning with the peak values of the stringency index. These declines might be attributed to people adhering to government strict stay-at-home orders, resulting in fewer vulnerable targets on the streets and a reduction of these crimes (which is aligned with RAT). Subsequently, the count began to rise once more, resuming the upward trend observed before the pandemic. This pattern suggests that the pandemic’s effect on the trend of violence and sexual offences was not enduring and the original trajectory persisted. According to Fig. 4(l), the pattern holds true for the majority of the local authorities. However, it is worth noting that, similar to public order crimes, several regions experienced peak levels of violence and sexual offences in 2018. In Fig. 5(l), we can observe the impact of the stringency index on violence and sexual offences across England and Wales. According to the figure, while the majority of local authorities experienced negative impact values, there are some local authorities in the middle and east regions of England that experienced positive impact values.

## Conclusions and Future Work

The security of society, economic progress, people’s daily routine, mental well-being, criminal activities, and more have all been profoundly affected by the unprecedented consequences of the recent COVID-19 pandemic across the globe. This study investigated the shifts in various categories of crime over a 9-year period from 2015 to 2023 for local authorities in England and Wales. It also considered the potential reasons behind these shifts and explored the impact of varying degrees of lockdown measures on different types of crime using a Bayesian spatio-temporal model.

The analysis of temporal patterns revealed that certain types of crimes in England and Wales underwent substantial alterations throughout the pandemic. These shifts were particularly pronounced when the stringency index was elevated, indicating greater constraints on citizens’ mobility. This observation aligns with criminological theories like the GST, RAT and SAT, which posit that external constraints and opportunities can impact criminal behaviour. Additionally, for certain types of crimes, the alterations in their trend diminished as pandemic measures were eased, returning to their pre-pandemic trajectories. However, as of the time of writing this paper, crime types such as burglary, robbery, and vehicle crime have not returned to their pre-pandemic levels. While the pandemic is a contributing factor to this ongoing change, there are other potential factors that may have contributed to the persistence of lower levels of these crimes, including increased occupancy of residences due to the adoption of remote work arrangements and heightened security measures implemented in both public and private spaces. We also discovered that contrary to the belief that the pandemic led to an increase in bicycle thefts, both the trend and the total count of this crime remained relatively stable and did not exhibit significant changes from the pre-pandemic trend and total counts. However, the only exception is London, where bicycle theft increased as a result of the pandemic. For shoplifting and theft from the person crimes, which were significantly influenced by business closures and restricted mobility, we observed a similar trend during the pandemic period. Both of these crime types witnessed a substantial decline initially, but as pandemic restrictions were eased and we approached the end of the pandemic, the incidences of these crimes began to rise, eventually returning to pre-pandemic levels. Although anti-social behaviour and drugs crimes differ significantly in nature, they both exhibited a significant increase at the onset of the pandemic. Subsequently, as pandemic restrictions were relaxed, both crime types experienced a resurgence, returning to their pre-pandemic levels. Each of these crime categories can be attributed to specific factors contributing to their sharp increases. For instance, the rise in drugs crime can be linked to a shift in law enforcement priorities, whereas the increase in anti-social behaviour crimes can be traced to dissatisfaction among certain individuals with government-imposed measures. The incidence of possession of weapons, public order, violence and sexual offences and criminal damage and arson declined during the pandemic. However, as the pandemic neared its conclusion, there was a resurgence in these offences, with their counts increasing and their trend returning to their pre-pandemic.

In terms of spatial pattern, our model’s findings, as illustrated in Fig. 5, indicate that the impact of the stringency index on anti-social behaviour and drugs crimes remained predominantly positive and consistent across local authorities in England and Wales. Conversely, this impact was predominantly negative and consistent for vehicle crime, burglary, shoplifting, criminal damage and arson, and theft from the person in the study area. For other types of crimes, the effect of the stringency index varied significantly across regions. This signifies that in certain local authorities, the effect was positive, while in others it was negative or insignificant.

This study’s findings highlight several key policy implications for maintaining public safety during future health crises. The observed reduction in crimes such as burglary and vehicle theft during strict lockdowns suggests that increased residential occupancy and mobility restrictions can effectively deter these types of criminal activities. Policymakers could consider promoting remote work and strengthening neighborhood security measures in similar situations. The increase in anti-social behavior and drug-related crimes during the pandemic underscores the importance of mental health support and community engagement. Law enforcement must strike a balance between enforcing public health measures and traditional crime prevention strategies, such as neighborhood watch programs, random stop-and-search operations, and foot patrols, to prevent unintended outcomes.

The varying impact of the stringency index across regions highlight the need for tailored, data-driven approaches. Customizing interventions to reflect local demographics and socioeconomic conditions can enhance the efficiency and effectiveness of resource allocation. Finally, since some crimes returned to pre-pandemic levels once restrictions were lifted, policymakers should prepare for post-crisis transitions by maintaining specific preventive measures and ensuring clear communication with the public.

There are some limitations to this work. First, we did not explore the underlying causes of the variation observed in the effect of the stringency index across different local authorities and types of crime. Several factors, including population density, demographics, and socioeconomic variables, might account for these variations (Kinney et al., 2008). Future research could utilize data associated with local authorities to examine the contribution of these factors to the spatial variations observed among them. Second, our study is limited by the incomplete data release from the Greater Manchester police force, which prevented us from incorporating data from local authorities within Greater Manchester. Third, our results, which include maps and heatmaps, reflect various events occurring across time and space. We were unable to discuss all of them in detail, but we believe there are interesting patterns that merit further investigation in future work. Lastly, investigating crime evolution at a finer spatial scale, such as the street level, through methods such as point pattern analysis could uncover localized variations and provide a deeper understanding of the crime dynamics before, during, and after the COVID-19 pandemic.

## Data Availability

We provide both the raw and final versions of the dataset used in our study. To ensure the reproducibility and transparency of our model and results, the code for running the analysis is available online at https://github.com/nilips70/Crime-Modelling. Crime data in its raw form is obtained from the UK Police website(https://data.police.uk/data/). The Government’s Response Oxford Stringency Index is extracted from the Our World in Data website(https://ourworldindata.org/coronavirus/country/united-kingdom). Lastly, the shapefile of UK local authorities’ boundaries is sourced from the Office for National Statistics website(https://geoportal.statistics.gov.uk/search?collection=Dataset). Details on how to process the data are provided in the repository.

## Footnotes

1 https://data.police.uk/data

2 https://ourworldindata.org/explorers/coronavirus-data-explorer?uniformYAxis=0&country=~GBR&hideControls=true&Interval=7-day+rolling+average&Relative+to+Population=true&Color+by+test+positivity=false&Metric=Stringency+index

3 https://www.bsg.ox.ac.uk/research/covid-19-government-response-tracker
